# Performance of nanoScan PET/CT and PET/MR for quantitative imaging of ^18^F and ^89^Zr as compared with ex vivo biodistribution in tumor-bearing mice

**DOI:** 10.1186/s13550-021-00799-2

**Published:** 2021-06-12

**Authors:** Marion Chomet, Maxime Schreurs, Ricardo Vos, Mariska Verlaan, Esther J. Kooijman, Alex J. Poot, Ronald Boellaard, Albert D. Windhorst, Guus AMS van Dongen, Danielle J. Vugts, Marc C. Huisman, Wissam Beaino

**Affiliations:** Amsterdam UMC, Vrije Universiteit Amsterdam, Radiology & Nuclear Medicine, Cancer Center Amsterdam, De Boelelaan 1117, Amsterdam 1081 HV, The Netherlands

**Keywords:** PET-CT, PET-MRI, Preclinical imaging, Quantification, Ex vivo biodistribution

## Abstract

**Introduction:**

The assessment of ex vivo biodistribution is the preferred method for quantification of radiotracers biodistribution in preclinical models, but is not in line with current ethics on animal research. PET imaging allows for noninvasive longitudinal evaluation of tracer distribution in the same animals, but systemic comparison with ex vivo biodistribution is lacking. Our aim was to evaluate the potential of preclinical PET imaging for accurate tracer quantification, especially in tumor models.

**Methods:**

NEMA NU 4-2008 phantoms were filled with ^11^C, ^68^Ga, ^18^F, or ^89^Zr solutions and scanned in Mediso nanoPET/CT and PET/MR scanners until decay. N87 tumor-bearing mice were i.v. injected with either [^18^F]FDG (~ 14 MBq), kept 50 min under anesthesia followed by imaging for 20 min, or with [^89^Zr]Zr-DFO-NCS-trastuzumab (~ 5 MBq) and imaged 3 days post-injection for 45 min. After PET acquisition, animals were killed and organs of interest were collected and measured in a *γ*-counter to determine tracer uptake levels. PET data were reconstructed using TeraTomo reconstruction algorithm with attenuation and scatter correction and regions of interest were drawn using Vivoquant software. PET imaging and ex vivo biodistribution were compared using Bland–Altman plots.

**Results:**

In phantoms, the highest recovery coefficient, thus the smallest partial volume effect, was obtained with ^18^F for both PET/CT and PET/MR. Recovery was slightly lower for ^11^C and ^89^Zr, while the lowest recovery was obtained with ^68^Ga in both scanners. In vivo, tumor uptake of the ^18^F- or ^89^Zr-labeled tracer proved to be similar irrespective whether quantified by either PET/CT and PET/MR or ex vivo biodistribution with average PET/ex vivo ratios of 0.8–0.9 and a deviation of 10% or less. Both methods appeared less congruent in the quantification of tracer uptake in healthy organs such as brain, kidney, and liver, and depended on the organ evaluated and the radionuclide used.

**Conclusions:**

Our study suggests that PET quantification of ^18^F- and ^89^Zr-labeled tracers is reliable for the evaluation of tumor uptake in preclinical models and a valuable alternative technique for ex vivo biodistribution. However, PET and ex vivo quantification require fully described experimental and analytical procedures for reliability and reproducibility.

**Supplementary Information:**

The online version contains supplementary material available at 10.1186/s13550-021-00799-2.

## Introduction

Preclinical positron emission tomography (PET) imaging has become a crucial tool for the development and evaluation of radiolabeled tracers and therapeutic drugs and can facilitate faster translation from bench to bedside [1, 2]. Preclinical PET cameras have been in constant evolution to offer improved spatial and temporal resolution and sensitivity. Nowadays, the advantages of functional and anatomical imaging are combined within hybrid cameras where computerized tomography (CT), magnetic resonance (MR) or fluorescent imaging technologies are added to the PET scanners [3, 4]. Compared to CT, MRI offers better soft tissue resolution and no radiation exposure. However, preclinical and clinical development of PET/MR scanners came with their own specific technical hurdles due to the strong magnetic field that might affect PET functionality, making the combination of the PET with MRI components more challenging than with CT. Furthermore, as MRI does not provide information on electron density, the attenuation coefficient map is more difficult to obtain and can result in lower imaging resolution [2].

To evaluate the potential and in vivo behavior of radiotracers, ex vivo biodistribution is generally considered the method of choice. In such studies, the radiotracer is administered to animals, and at predetermined time points tissues of interest are collected, weighed, and counted for radioactivity. Percentage of injected dose (or percentage of activity) per gram of tissue (%ID/g or %IA/g) is then calculated providing information about the biodistribution and in vivo kinetics of the PET tracer. A large number of animals is usually used for a limited number of time points, which is not in line with the ethical aim to reduce animal use. Unlike ex vivo biodistribution, preclinical PET imaging is offering the possibility for longitudinal studies, where the same animals can be used as their own control at multiple and early time points. This fully complies with current ethics on animal experiments and the 3Rs guidelines (Reduction, Replacement, Refinement) [5–8]. Furthermore, PET imaging allows quantitative and visual whole body assessment of tracer uptake giving insights on the heterogeneity of its distribution often not possible with ex vivo biodistribution. Despite the increased availability of preclinical PET cameras in research centers, PET imaging is mostly used as a qualitative visualization tool and not as a replacement for ex vivo quantification of biodistribution [9]. The review of Kuntner and Stout [10] summarized the crucial parameters that are needed to obtain reliable and reproducible quantitative PET data in small animals studies: i. the camera itself (i.e., size, type, and number of detectors), ii. the physical properties of the PET isotope used (i.e., positron range), and iii. the animal models and animal handling. Moreover, preclinical PET studies still lack standardized protocols that lead to unreliable data making inter-study comparison more challenging.

In recent publications, the reliability and reproducibility of preclinical data acquired using phantoms and various cameras in different imaging centers were evaluated. The key factors that resulted in variability were, apart from the type of camera used, the differences in animal handling and image acquisition protocols and analysis, and variability between users [11–13].

PET imaging is rarely used for the quantification of radiotracer biodistribution and data that allow comparison between in vivo and ex vivo uptake are very scarce. The influence of the partial volume effect (PVE) is often mentioned as a cause of quantification inaccuracy. PVE results from the limited spatial resolution of preclinical cameras which impairs accurate measurement of activity concentrations in small regions surrounded by other organs or background activity. Consequently, an underestimation of tracer uptake, especially in, e.g., small tumors, orthotopic or metastatic models, organs or brain sub-regions is observed [10].

In this study, we evaluated the performance of a Mediso nanoScan PET/CT and PET/MR scanner for tracer quantification in subcutaneous tumors and selected organs by direct comparison of PET imaging with ex vivo biodistribution. We evaluated the critical parameters involved in reliable PET quantification in vitro as well as in vivo. Firstly, phantoms filled with the most commonly used PET radionuclides ^11^C, ^68^Ga, ^18^F, and ^89^Zr were used in order to evaluate the performance of the cameras with respect to linearity, recovery coefficient, PVE and spill-over effect. Secondly, breast cancer tumor-bearing mice were used for quantification of [^18^F]FDG and [^89^Zr]Zr-DFO-NCS-trastuzumab uptake in tumors and selected organs (brain, kidney and liver) by PET imaging as well as by ex vivo biodistribution. Congruency between PET quantification and ex vivo biodistribution was evaluated using Bland–Altman plots.

## Materials and methods

### Phantom experiments

All experiments were performed with a Mediso nanoScan PET/CT and Mediso nanoScan PET/MR (Mediso Ltd., Hungary) [14]. Two NEMA NU 4–2008 phantoms, in conditions similar to those described by others [15–18], were filled with solutions containing either carbon-11 (*t*_1/2_ = 20.4 min, *I*_*ß*+_ = 100%, *E*_*ß*max_ = 960 keV, [^11^C]acetate), gallium-68 (*t*_1/2_ = 67.6 min, *I*_*ß*+_ = 90%, *E*_*ß*max_ = 1900 keV, [^68^Ga]GaCl_3_ in 0.1 M HCl), fluorine-18 (*t*_1/2_ = 109.8 min, *I*_*ß*+_ = 100%, *E*_*ß*max_ = 634 keV, [^18^F]FDG) or zirconium-89 (*t*_1/2_ = 78.4 h, *I*_*ß*+_ = 23%, *E*_*ß*max_ = 897 keV, [^89^Zr]Zr-DFO-NCS-trastuzumab). [^11^C]acetate and [^89^Zr]Zr-DFO-NCS-trastuzumab were produced in house, the latter as described by Vugts et al. [19], making use of [^11^C]CO_2_ or [^89^Zr]Zr-oxalate in 1 M oxalic acid (Perkin-Elmer, Boston, USA). [^18^F]FDG was purchased from Cyclotron BV and [^68^Ga]GaCl_3_ was provided by the department of radiology and nuclear medicine of the Academic Medical Center (AMC), Amsterdam UMC, the Netherlands. Linearity, reproducibility, spill-over effects, and recovery coefficients (RC) were determined for each camera. Regions of interest (ROIs) in the phantom cylinders were drawn with sizes matching the contours of the cylinders (1–5 mm). This decision was made for practical reasons to closely match the in vivo tumor analysis strategy where ROIs are drawn on the actual sizes of the tumor. As a second approach, ROI analysis was performed according to the standardized NEMA protocol [17] as a generic quality control using the automatic tool from the Mediso quality control software provided with the cameras in which the ROI sizes are twice the actual size of the cylinders. Detailed description of the phantom experiments can be found in Additional file 1.

### Animal experiments

#### Animals

Animal experiments were performed in accordance with the European Community Council Directive (2010/63/EU) for laboratory animal care and the Dutch Law on animal experimentation and following the ARRIVE guidelines 2.0 [20]. The experimental protocol was validated and approved by the central committee for animal experimentation (CCD) and the local committee on animal experimentation of the VU University Medical Center. Mice were housed under standard laboratory conditions with water and food ad libitum. Study design and sample size: A well-known strain of mice and cell line were chosen for the evaluation of tracers biodistribution and tumor uptake. Athymic nude Foxn1^nu/nu^ mice (*n* = 30, 8 weeks old, 18–25 g, Envigo, Horst, The Netherlands) were injected subcutaneously (s.c.) in both flanks with 2.5 × 10^6^ HER2-positive N87 human breast cancer cells (American Type Culture Collection (ATCC)). Tumor size was measured with a caliper ((Length × Width × Depth)/2) and tracers were injected when the average size reached 100 mm^3^. 10 mice per group were used to ensure a sufficient number of animals for reliable analysis. In the ^18^F study (*n* = 20), animals were imaged by PET/CT (*n* = 10) or by PET/MR (*n* = 10) followed by ex vivo biodistribution. In the ^89^Zr study (*n* = 10), animals were imaged by PET/CT and PET/MR before ex vivo biodistribution. Inclusion and exclusion criteria: time points were decided a priori and correspond to standard practice with the ^18^F and ^89^Zr tracers used. No data were excluded from analysis except obvious outliers (i.e., organ out of field of view during data acquisition, see results). Randomization: In the ^18^F study, mice were attributed randomly per camera. Outcome: Quantification of PET imaging and ex vivo biodistribution was performed for all animals. The comparison between the quantification data derived from PET imaging and ex vivo biodistribution in tumors was the main study goal.

#### Scan protocol

For the ^18^F study, tumor-bearing mice (*n* = 20) were intravenously (i.v.) injected through the tail vein with [^18^F]FDG (120–200 µL diluted in 0.9% NaCl, 14.3 ± 0.7 MBq). Injections were performed under anesthesia (2–4% isoflurane/O_2_). Animals were kept under anesthesia on a heated pad for 50 min, followed by acquisition of the PET image for 20 min in the PET/CT (*n* = 10 mice) or the PET/MR (*n* = 10 mice) scanner. CT and MR scans were acquired just before the start of the PET acquisition. Post-PET imaging, the animals were immediately killed for ex vivo biodistribution. For the ^89^Zr study, tumor-bearing mice (*n* = 10) were injected i.v. in the tail vein with 100 µg of the HER2 targeting antibody [^89^Zr]Zr-DFO-NCS-trastuzumab (117 ± 8 µL, 5.4 ± 0.2 MBq). PET scans were acquired for 45 min at 72 h p.i.. The mice were first scanned in the PET-CT, directly followed by scanning in the PET/MR. While being still under anesthesia, mice were killed by cervical dislocation directly after the last PET scan for assessment of ex vivo biodistribution.

#### Ex vivo biodistribution

Blood, tumors, and organs of interest were collected, weighted and the amount of radioactivity in each sample was measured in the same *γ*-counter as described in the phantom experiments (Additional file 1). During organ collection, attention was paid, i.e., for tumors to remove fat, skin or other surrounding tissues. Organs were collected entirely (i.e., brain, kidney) except for big organs such as liver for which one lobe was collected. Organs collected during the ^18^F study were immediately counted in the *γ*-counter after dissection. Organs collected in the ^89^Zr study were counted up to 2 weeks after imaging due to the long physical half-life of ^89^Zr and the high level of activity injected. To limit geometric effects, 1 mL of water was added to all tubes containing the collected organs before counting in the γ-counter. The total dose injected per animal was either determined by measuring syringes before and after injection in the dose calibrator (^18^F study) or determined based on weighted standards (8 standards of 10 µL) from the same radiotracer solution as administered to the animals (^89^Zr study). Radioactivity uptake in each organ was calculated as the percentage of the injected activity per gram of tissue (%IA/g) and decay-corrected to the time of PET imaging. The term %IA/g is further used in the rest of the manuscript as a more appropriate terminology than injected dose per gram of tissue (%ID/g).

#### PET-assessed biodistribution

Reconstruction of static PET images was performed using the fully three-dimensional reconstruction algorithm (Tera-TomoTM, Mediso Ltd.) with 4 iterations and 6 subsets, and an isotropic 0.4 mm voxel dimension recommended by Mediso Ltd as default reconstruction algorithm for animal studies. PET image analysis was performed using VivoQuant^®^ 3.5 software (Invicro). Tumor (left and right), and left kidney ROIs were manually drawn plane by plane following CT or MR anatomical delineation of the organ. Brain ROI was obtained using the VivoQuant brain atlas tool. Liver uptake was obtained by drawing a sphere-shaped ROI into the left liver lobe, corresponding with the lobe counted for assessment of ex vivo biodistribution. The image-derived total activity was obtained by drawing a ROI around the whole PET image 3D volume. Kidney, brain and liver were chosen because the contrast in the CT and MR images allows accurate delineation of the ROIs. These organs can also be dissected for biodistribution without contamination from other tissues. Mean activities in Bq/mL were extracted from ROIs and used for all calculations. Tissue tracer concentration in Bq/mL was converted to %IA/g, decay-corrected to time of injection, and corrected using the average cross-calibration (CC) factors between the camera and the dose calibrator (see Additional file 1) to allow comparison with the ex vivo biodistribution results.

### Analysis

In the ^18^F in vivo study, PET imaging was performed for 20 min prior biodistribution. PET scans (*n* = 3 mice) were reconstructed in four frames of 5 min and tumor quantification was compared to the 20 min reconstructed frame to investigate potential tracer elimination during the scanning acquisition time. Data revealed minimal differences in uptake values assuring validity of direct comparison of PET imaging quantification to the biodistribution.

Quantification data derived from the PET images and ex vivo biodistribution were compared using Bland–Altman plots [21, 22]. Bland–Altman plots are obtained by plotting the ratio PET/ex vivo assessed uptake on the *Y*-axis, and the average uptake (in %IA/g) of both quantification methods used (PET and biodistribution) on the *X*-axis, resulting in the mean of the ratios, i.e., the bias ± SD. For example, a bias of 0.88 ± 0.11 means that on average PET assessed uptake is 12% lower than ex vivo assessed uptake with in this case 11% standard deviation (sd). 95% limits of agreement represented in Bland–Altman plots are obtained from “bias ± 1.96 × sd”. Graphs and figures were made using GraphPad Prism version 8.2.1 (San Diego, CA, USA).

## Results

### Phantom experiments

**Fig. 1:**
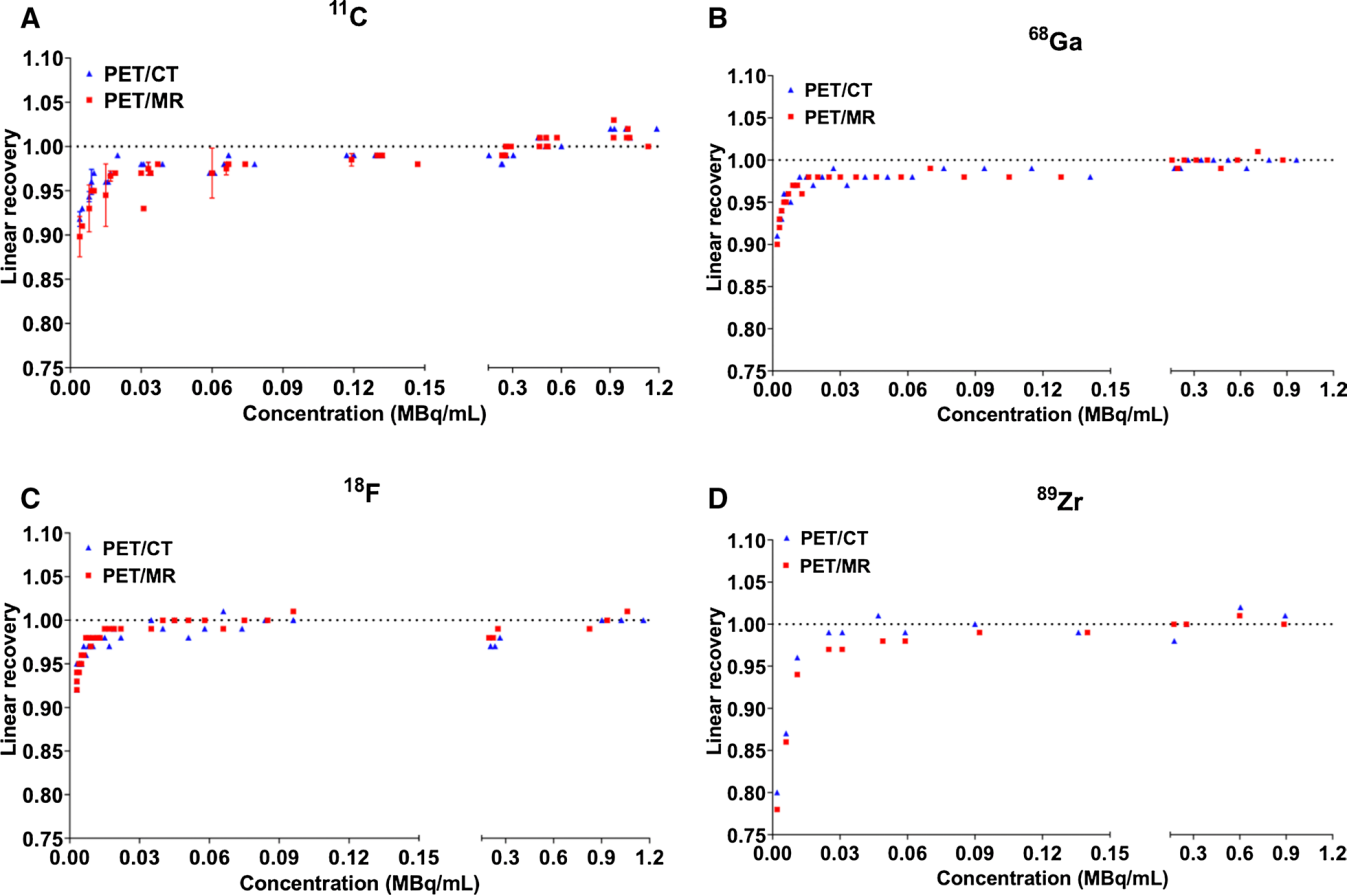
Linear recovery of the PET/CT and PET/MR scanners as assessed with ^11^C (n = 6 phantoms) (**A**), ^68^Ga (**B**), ^18^F (**C**) and ^89^Zr (**D**). Note the impaired linearity at doses < 0.05 MBq/mL for each of the radionuclides

Cross-calibration factors between the dose calibrator and the two cameras are presented in Additional file 1: Table S1. Linear recovery of the activity was achieved by the PET/CT and PET/MR for all the tested activity concentration range until around 0.05 MBq/mL (Fig. 1).

**Fig. 2:**
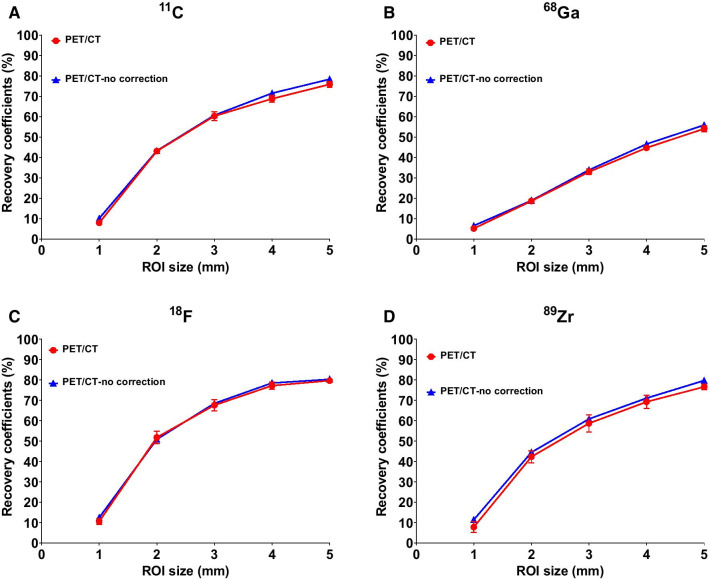
NanoScan PET/CT recovery coefficients for ^11^C (**A**), ^68^Ga (**B**), ^18^F (**C**), and ^89^Zr (**D**) with and without scatter and attenuation correction (see also Additional file 1: Table S2) using the TeraTomo reconstruction algorithm

Recovery coefficients (RCs) determined for the PET/CT for all isotopes are reported in Fig. 2 and Additional file 1: Table S2. The highest recovery coefficient (80%), and thus the lowest PVE was obtained for ^18^F in the 5 mm rod while ^11^C and ^89^Zr showed a slightly lower recovery of 76% and 77%, respectively. ^68^Ga had the lowest recovery coefficient of 54% in this 5 mm rod. PET/MR showed similar RCs as the PET/CT. RCs according to the standardized NEMA protocol at two representative doses of 3.7 and 20 MBq in the PET/CT are presented in Additional file 1: Figure S1. Spill-over effects in air and water ranged between 12 and 17% for all isotopes and both cameras except for ^68^Ga (> 20% in water) (Additional file 1: Figure S2).

### Animal experiments

Total injected activity derived from PET imaging by whole animal ROI accurately matched with the total activity determined by the dose calibrator. The ratio between PET/ [^18^F]FDG injected activity was 1.03 ± 0.04 for the PET/CT and 1.01 ± 0.08 for the PET/MR. Ex vivo biodistribution results for [^18^F]FDG and [^89^Zr]Zr-DFO-NCS-trastuzumab are presented in Additional file 1: Table S3. The tumor size determined by PET ROIs and weights during biodistribution are summarized in Additional file 1: Table S4. The overall correlation between image assessed tumor size and ex vivo tumor weights was high with *R*^2^ = 0.90 and 0.88 in the ^18^F study for the PET/CT and PET/MR mice groups, respectively. Those ratios were slightly lower in the ^89^Zr study, where the average tumor size was smaller than in the ^18^F study, with *R*^2^ of 0.84 and 0.75 when the volume was assessed using the PET/CT and the PET/MR, respectively (same group of mice were imaged in both cameras before ex vivo biodistribution).

#### Biodistribution: uptake in tumors

**Fig. 3:**
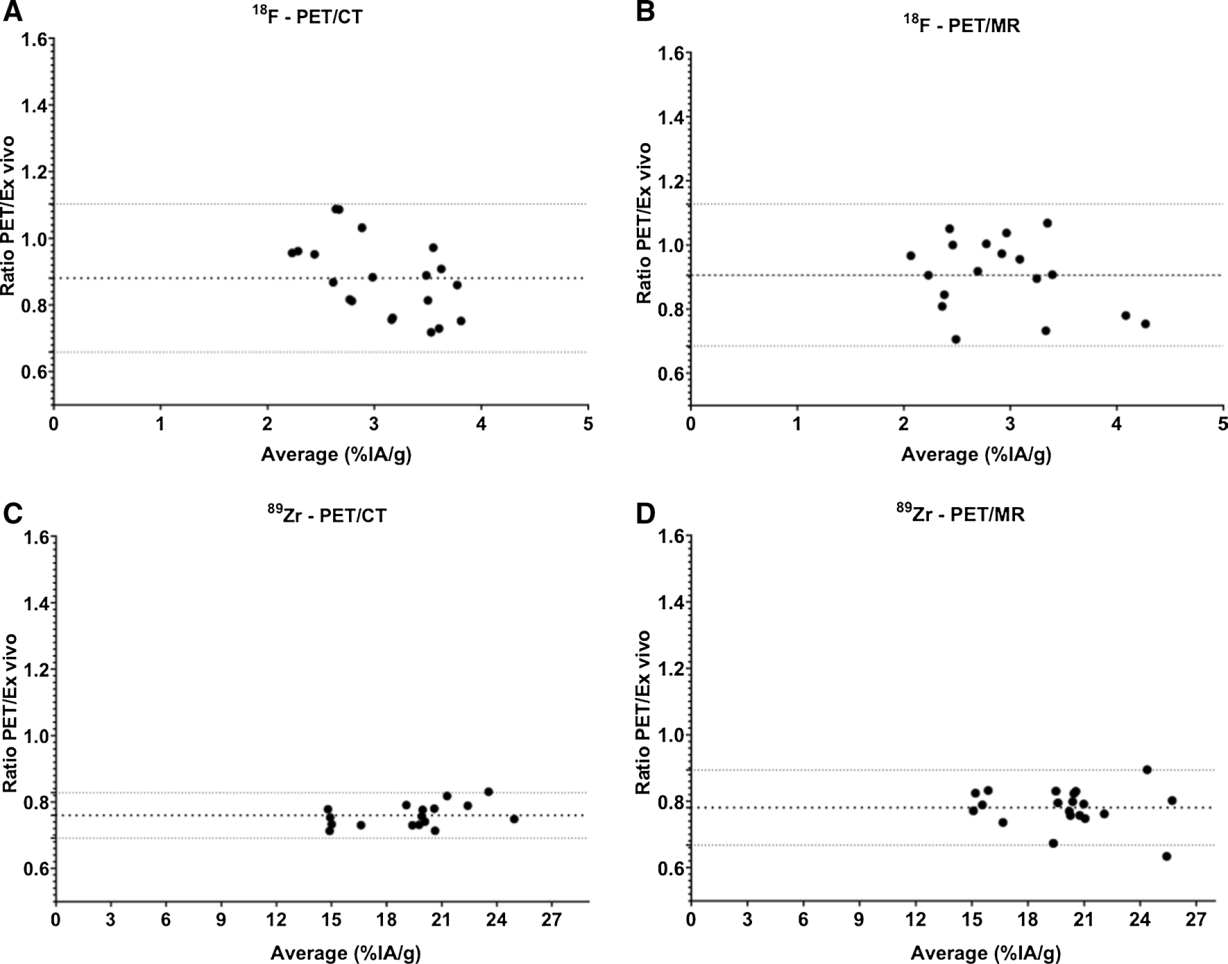
Bland–Altman plots comparing tumor uptake of [^18^F]FDG (**A**, **B**) and [^89^Zr]Zr-DFO-NCS-trastuzumab (**C**, **D**) assessed by PET imaging (PET/CT: **A**, **C**; PET/MR: **B**, **D**) or by ex vivo biodistribution. The middle-dotted line shows the Bias (mean of the ratios) and the upper and lower dotted lines show the 95% limits of agreement. Average (%IA/g) corresponds to the average uptake value per animal between PET and ex vivo biodistribution

**Fig. 4:**
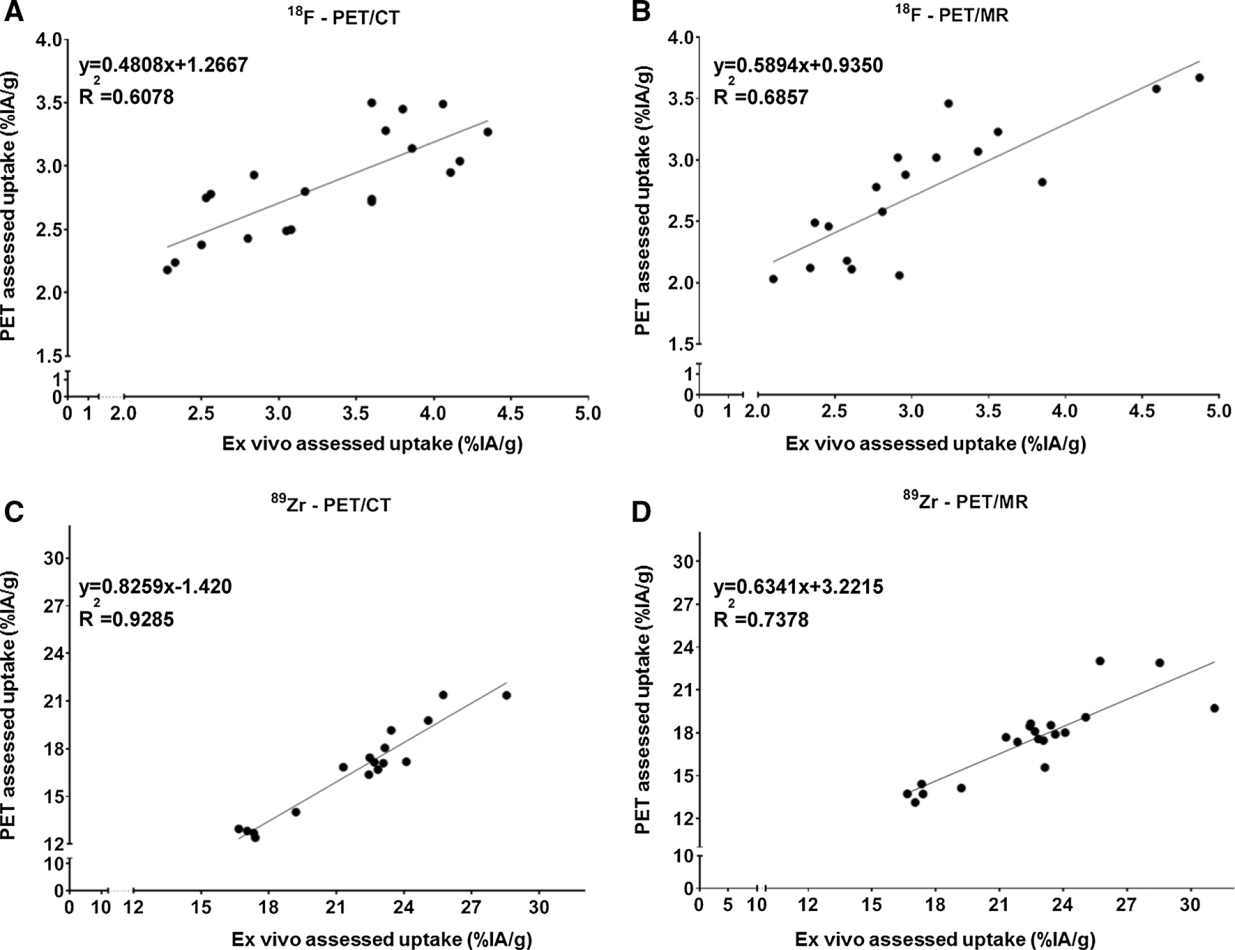
Linear regression plots showing the correlation between tumor uptake assessed by PET imaging (PET/CT: **A**, **C**; PET/MR: **B**, **D**) and ex vivo biodistribution for [^18^F]FDG (**A**, **B**) and [^89^Zr]Zr-DFO-NCS-trastuzumab (**C**, **D**)

Bland–Altman plots and linear regression analyses of tracer uptake in tumors are presented in Figs. 3 and 4, and in Table 1 and are derived from the individual results of each mouse reported in Additional file 1: Table S5.

**Table 1:** Bland–Altman results for the comparison of tracer uptake quantification (%IA/g) assessed by PET imaging and by ex vivo biodistribution for [^18^F]FDG and [^89^Zr]Zr-DFO-NCS-trastuzumab

		PET/CT		PET/MR	
		^18^F	^89^Zr	^18^F	^89^Zr
Tumor	Bias ± sd	0.88 ± 0.11	0.76 ± 0.03	0.91 ± 0.11	0.78 ± 0.06
Brain	Bias ± sd	1.07 ± 0.03	2.89 ± 0.46^a^	1.03 ± 0.10	3.54 ± 0.82^a^
Kidney	Bias ± sd	0.85 ± 0.11	0.86 ± 0.10	0.89 ± 0.11	0.92 ± 0.10
Liver	Bias ± sd	1.22 ± 0.09	1.06 ± 0.05	1.22 ± 0.15	1.15 ± 0.07

^a^Large bias due to denominator of the ratios close to 0%IA/g

In the [^18^F]FDG study, tumor uptake in the two groups as assessed by PET/CT and PET/MR was comparable (2.9 ± 0.4%IA/g and 2.8 ± 0.5%IA/g, respectively, Additional file 1: Table S5) but showed slightly lower uptake compared to ex vivo biodistribution (3.3 ± 0.7 and 3.1 ± 0.7%IA/g, respectively) (Additional file 1: Table S3). Similar observations were made in the ^89^Zr study: PET/CT showed 16.7 ± 2.9%IA/g of [^89^Zr]Zr-DFO-NCS-trastuzumab in tumor and PET/MRI 17.5 ± 2.7%IA/g, while on average uptake according to ex vivo biodistribution was 22.5 ± 3.7%IA/g. In the ^18^F study, the bias (PET/ex vivo) was 0.88 ± 0.11 and 0.91 ± 0.11 for the PET/CT and PET/MR, respectively, while the 95% agreement interval was relatively large with 0.66–1.10 for PET/CT and 0.68–1.13 for PET/MR (Fig. 3A, B and Table 1). In the [^89^Zr]Zr-DFO-NCS-trastuzumab study, the bias PET/ex vivo was slightly worse (0.76 ± 0.03 for PET/CT and 0.78 ± 0.06 for PET/MR). The standard deviation between the quantification methods was however small with a 95% agreement interval of 0.69–0.83 for PET/CT and 0.67–0.89 for PET/MR (Fig. 3C, D and Table 1). This observation was in accordance with the *R*^2^ values of the linear regression showing a good agreement between the uptake assessed by PET and ex vivo biodistribution (Fig. 4).

We also observed a small contribution of the PVE in the PET quantification of the smaller tumors that resulted in a lower PET/ex vivo ratio (Additional file 1: Figure S3).

#### Biodistribution: uptake in selected organs

Bland–Altman analyses of tracer uptake in brain, kidney and liver are shown in Table 1 and Additional file 1: Figures S4–S6 and are derived from the individual results of each mouse reported in Additional file 1: Table S5.

For the brain, [^18^F]FDG uptake assessed by PET/CT showed a PET/ex vivo bias of 1.07 ± 0.03 with a very narrow 95% agreement interval (1.01–1.13). With the PET/MR, the bias was closely matching the PET/CT results (1.03 ± 0.10), but with a larger 95% agreement interval (0.83–1.23) (Table 1, Additional file 1: Figure S4A, B). In the ^89^Zr study, brain uptake of the monoclonal antibody trastuzumab was as expected very low with an average uptake between the two quantification methods of ~ 0.4–0.7%IA/g (Additional file 1: Figure S4C, D) close to background. Therefore, variations between the two quantification methods were large. Overall, PET derived uptake was higher than uptake determined by ex vivo biodistribution (bias > > 1; Table 1, Additional file 1: Figure S4C, D). In kidney, very similar overall bias and 95% agreement interval were obtained with both cameras in both ^18^F and ^89^Zr studies (bias ~ 0.9 ± 0.1) (Table 1, Additional file 1: Figure S5). Finally, liver results showed a bias of 1.22 ± 0.09 and 1.22 ± 0.15 in the ^18^F study and 1.06 ± 0.05 and 1.15 ± 0.07 in the ^89^Zr study for PET/CT and PET/MR, respectively (Table 1, Additional file 1: Figure S6).

## Discussion

In this study, we evaluated the performance of preclinical PET imaging quantification versus ex vivo biodistribution to assess whether and under which conditions PET imaging is able to replace ex vivo biodistribution. This is of particular interest in longitudinal studies with xenograft-bearing mice where accurate tumor uptake quantification via PET imaging would drastically reduce the number of animals used for tracer evaluation. For this purpose, we compared the uptake derived from the PET/CT and the PET/MR images with ex vivo biodistribution in N87 tumor-bearing mice with the most commonly used PET radionuclide, ^18^F (as in [^18^F]FDG). To further compare both quantification methods, we evaluated in the same animal model, mice injected with a long-lived radionuclide matching the biological half-life time of monoclonal antibodies: ^89^Zr as in [^89^Zr]Zr-DFO-NCS-trastuzumab, a well-known HER2 targeting monoclonal antibody.

Before performing in vivo studies, we evaluated the nanoScan PET/CT and PET/MR using the preclinical NEMA NU 4-2008 phantom in a standard approach and obtained results in line with others (Figs. 1, 2 and Additional file 1: S1–S2) [12, 14, 16, 18, 23]. ^18^F outperformed all radionuclides with the highest recovery coefficient (80%), while ^11^C and ^89^Zr had a lower but comparable recovery and ^68^Ga the lowest (54%) as could be expected from physical properties, *ß*^+^ energies and range in water, of those radionuclides [24]. In the case of ^18^F and ^89^Zr, further explored in the in vivo studies, there are many reasons for lower RCs for ^89^Zr in comparison with ^18^F including the following: (i) ^89^Zr possesses a lower signal to noise ratio compared to ^18^F which can be due to lower injected activities and the fact that ^89^Zr has a lower positron branching fraction (23%) compared to ^18^F (97%), (ii) the positron range of ^89^Zr is larger than ^18^F (respective mean range in water 1.2 and 0.6 mm) leading to a lower effective spatial resolution and thus lower RCs [16].

The unique aspect of the phantom studies apart from evaluating both a PET/CT and PET/MR from the same provider with four radionuclides (^11^C, ^68^Ga, ^18^F and ^89^Zr) was that we evaluated the RCs in the five cylinders of the phantom based on ROIs sizes matching the real contours of the cylinders (Fig. 2) and not only using ROI sizes twice their actual size as is performed in the standard NEMA quality control of the cameras (as represented in Additional file 1: Figure S1). This explains why RCs in Additional file 1: Figure S1 were higher than in Fig. 2. Our method was intended to reflect the approach used later in vivo where tumors were also delineated based on the exact contours of the organ.

The number of organs that can accurately be segmented and thus quantified with PET imaging is more limited than with ex vivo biodistribution. Even though subcutaneous tumor xenograft can usually be very well delineated, they may present various shapes and few studies have been comparing tumor uptake derived from PET imaging directly to ex vivo biodistribution. As tumors were the main organ of interest, they were delineated based on CT or MR to define ROIs and this method proved indeed to be reliable.

To obtain ROIs, we defined a systematic approach without the use of (semi-) quantifying tools for all organs to be able to compare with ex vivo biodistribution. The eventual choice of predefined quantifying tools should always be well justified as they can lead to large differences in assessed uptake that can limit inter-study comparisons. In our systematic approach, in comparison with ex vivo biodistribution, such tools were thus not used. Available tools for PET quantification and their influence on assessed uptake in organs have been nicely explored in the comprehensive paper from Mannheim et al. [11]. As an example, they compared [^18^F]FDG uptake in left ventricles by two different persons using fixed but different thresholding strategies, and this led to different uptake values, proving that this was not a reliable analysis method.

The correlation between tumor volume derived from organ delineation based on CT or MR and tumor weight assessed during biodistribution was high (*R*^2^ > 0.8 for all possible combinations, (tracer and cameras)) showing that the two methods of assessing the tumor weight are comparable and minimally affecting the %IA/g values. PET assessed uptake in tumors (in %IA/g) was consistently lower than ex vivo determined uptake with comparable biases for ^18^F and ^89^Zr for both cameras (Table 1). Underestimation of the PET in comparison with ex vivo biodistribution has been previously reported, using different systems and analysis methods and was attributed mostly to the inherent limited resolution of the systems, variation between ROI delineation methods and PVE [25, 26]. Tatsumi et al. [27] observed a recovery ratio of 80 ± 20% between PET/CT and ex vivo assessed activity concentrations (in Bq/mL) in tumor-bearing rats injected with [^18^F]FDG. In brain and kidney, they reported a lower recovery of 40 and 60%, respectively, and they attributed it to the smaller organ sizes compared with tumors.

In our study, the bias for the ratio PET/ex vivo in tumors was slightly lower for ^89^Zr compared to ^18^F which could be explained by the lower recovery coefficient of ^89^Zr compared to ^18^F caused by the difference in physical properties of both radionuclides (see before). However, 95% limits of agreement were better in the ^89^Zr study which could be due to the relatively high uptake of the ^89^Zr tracer in N87 tumors (~ 15–25%IA/g) when compared to [^18^F]FDG (~ 2–4%IA/g). Only a tendency for a lower PET/ex vivo uptake ratio with smaller tumors was observed in the ^18^F study with the PET/CT (Additional file 1: Figure S3A), and thus PVE was not identified to influence results in tumors.

An excellent bias for the PET/ex vivo ratios was obtained for the brain with the animals injected with [^18^F]-FDG, and a very narrow 95% agreement interval for the PET/CT (Table 1). These results suggest that the brain is a suitable organ for quantitative imaging of ^18^F tracers like [^18^F]FDG. However, this was not the case for ^89^Zr-radiolabeled-mAb, trastuzumab, where a high discrepancy between the two quantification methods was observed. This is likely attributed to the fact that the level of brain penetration of ^89^Zr-labeled mAbs is low (close to ~ 0%IA/g) with a lack of specific targeting, resulting in low organ to background ratios and relatively large biases of the ratios due to the small values. This aspect should be taken in consideration when evaluating antibodies for brain targeting [28].

For PET quantification, the lack of standardized protocols and the variability between animal handling, analysis and users has been reported recently [11]. In addition, our results suggest that quantification reliability also depends on the particular organ that is analyzed and how users define, select, and draw ROIs. For example, kidney and tumor were in our study always drawn based on anatomical images (CT or MRI) and not based on the PET signal, which in our opinion increases variability due to visual artefacts and scaling bias of the PET image. The soft tissue resolution of MR is better than CT allowing better delineation of organs that could influence the quantitative data derived from PET images. The overall comparison of tracer quantification by both cameras with ex vivo biodistribution especially in tumors gave very similar results suggesting that tumor delineation on CT is equally good as MR. In addition, the difference in attenuation and scatter correction algorithms in both scanners did not significantly affect the quality of the data [14].

To the best of our knowledge no preclinical study compared PET/CT and PET/MR scanners with ex vivo biodistribution for various radionuclides. This is most probably due to the fact that preclinical PET/MR systems were introduced recently in comparison with single PET and PET/CT scanners. Most studies in the past have estimated the outcome of in vivo studies solely based on the performance of the PET in phantoms which does not always predict the performance in animals [14, 18, 29–31].

It is also important to note that while ex vivo biodistribution is considered the gold standard for quantifying tracer biodistribution, in practice this technique might appear not perfectly standardized leading to different results between research centers. Technical details on how biodistribution is performed are often missing in publications, while this is important for standardized sampling. Critical details to be reported: is blood removed from tissues, is the organ collected entirely, and are fat, skin or other surrounding tissues properly removed? Next to this also the counting of radioactivity should be standardized, e.g., taking care of geometric effects and counting saturation (dead time).

Our manuscript described real-life issues related to uptake quantification and offers solutions on how to perform preclinical studies in a systematic way. Furthermore, this study provides a direct comparison of ex vivo biodistribution with PET/CT and PET/MR cameras from the same provider. PET appears to be a reliable quantification method to assess tumor uptake in xenografted mice, for which on top of the aforementioned parameters, an evaluation per organ and per radiotracer is necessary for future preclinical studies and comparison between them.

## Conclusion

In this study we compared preclinical PET imaging quantification with ex vivo biodistribution side by side in order to assess the potential of PET imaging as a replacement tool. Our study showed that both methods give comparable results within certain limits that are depending on the organ evaluated and radioisotope used. This is a step forward in validating the use of quantitative preclinical PET imaging for the assessment of tracer biodistribution. More studies evaluating different PET isotopes and organs of interest in different models of pathologies are needed to further assess the potential of PET imaging as substitute for ex vivo biodistribution. Improvement in PET quantification might still require advancement of the scanners themselves and their reconstruction algorithms. In addition, detailed and descriptive protocols should be reported in literature to ensure reliability and reproducibility of results.

## Supplementary Information

**Additional file 1.** Additional information on phantoms experiments, supplementary tables and figures regarding phantom experiments and *in vivo* studies.

## Data Availability

Not applicable.

## Abbreviations

PET: Positron emission tomography
CT: Computerized tomography
MR: Magnetic resonance
PVE: Partial volume effect
ROI: Region of interest
CC: Cross-calibration
%IA/g: Injected activity per gram of tissue
%ID/g: Injected dose per gram of tissue
sd: Standard deviation
RC: Recovery coefficient
