# Supplementary material for: Performance of nanoScan PET/CT and PET/MR for quantitative imaging of 18F and 89Zr as compared with ex vivo biodistribution in tumor-bearing mice
Source: EJNMMI Res. 2021 Jun 12;11:57. doi: 10.1186/s13550-021-00799-2 (PMC8197690; doi:10.1186/s13550-021-00799-2)
Supplement: Supplementary file 1 — Additional file 1. Additional information on phantoms experiments, supplementary tables and figures regarding phantom experiments and in vivo studies. [file 13550_2021_799_MOESM1_ESM.docx]

**Performance of nanoScan PET/CT and PET/MR for quantitative imaging of ^18^F and ^89^Zr as compared with *ex vivo* biodistribution in tumor bearing mice**

Marion Chomet^1^, Maxime Schreurs^1^, Ricardo Vos^1^, Mariska Verlaan^1^, Esther J. Kooijman^1^, Alex J. Poot^1^, Ronald Boellaard^1^, Albert D. Windhorst^1^, Guus AMS. van Dongen^1^, Danielle J. Vugts^1^, Marc Huisman^1^, Wissam Beaino^1^.

^1^Amsterdam UMC, Vrije Universiteit Amsterdam, Radiology and Nuclear Medicine, Cancer Center Amsterdam, De Boelelaan 1117, Amsterdam, The Netherlands.

**Keywords**

PET-CT, PET-MRI, preclinical imaging, quantification, *ex vivo* biodistribution

**First Author:** m.chomet@amsterdamumc.nl

**Corresponding Author:** w.beaino@amsterdamumc.nl

**Supplementary materials**

**MATERIALS AND METHOD**

**Phantom experiments**

The NEMA NU 4-2008 phantom consists of a main chamber, five fillable cylinders of 1, 2, 3, 4, or 5 mm diameter, and two cylindrical compartments filled with air and water. Radioactive solutions were prepared in glass vials (24 mL), and further diluted to fill the phantoms, with water in case of ^18^F and ^11^C solutions or with a solution of 40% 0.5 M HEPES (Invitrogen), pH 7 / 60% PBS containing 2% bovine serum albumin (Sigma-Aldrich) to avoid stickiness in case of [^89^Zr]Zr-DFO-NCS-trastuzumab and Gallium-68 (*1*). The radioactivity concentration (MBq/mL) was determined based on weight and radioactivity measurement in a dose calibrator (VDC 404, Veenstra, The Netherlands). This dose calibrator is periodically cross-calibrated for each radionuclide with the PET scanners and with the gamma counters (Wallac LKB-gamma counter 1282; Pharmacia) using a known amount of radioactivity to obtain CPM/Bq cross calibration (CC) factors. The phantom was filled using a 50 mL syringe with an average of 20 MBq of the corresponding radioactive solutions, and the exact activity in the phantom was determined based on the difference in weight before and after filling.

For ^11^C, ^68^Ga, ^18^F and ^89^Zr PET data were acquired starting from ~20 MBq until complete radioactive decay (less than 0.005 MBq/mL remaining). Due to the longer half-life of ^89^Zr, the phantom was scanned multiple times over a period of two weeks. Reconstruction with both scanners was performed using a fully 3-dimensional reconstruction algorithm (Tera-TomoTM, Mediso Ltd.) with scatter and attenuation correction, 4 iterations and 6 subsets, and an isotropic 0.4 mm voxel dimension. Data were analyzed with the Amide software (GNU General Public License, Version 2, Made.exe 0.9.2) and Regions of Interest (ROIs) were drawn as 3D cylinders. All radioactivity counts were decay corrected to the beginning of scanning. To assess linearity of scanning for both scanners, the total activity (Bq) in the phantom was determined over time by drawing a cylindrical ROI (40 x 40 x 60 mm) in the main body of the phantom and decay corrected. Reproducibility was assessed by repeating six times the phantom experiment filled with ^11^C.

To determine uniformity, a “uniformity” ROI (24 x 24 x 10 mm) was drawn in the main body of the phantom and the RC was calculated as: RC = (uniformity concentration (mean Bq/mL) x phantom volume (mL))/(total activity in the phantom (Bq))$.$

Partial volume effect (PVE) was estimated via recovery coefficients using the concentrations (mean Bq/mL) recovered in the five cylinders ROIs (5 x 5 x 5, 4 x 4 x 5, 3 x 3 x 5, 2 x 2 x 5, 1 x 1 x 5 mm) as a ratio compared against the concentration (mean Bq/mL) in the uniformity ROI.

Finally, spill-over effects in air and water were assessed using the corresponding air and water chambers (7.9 x 7.9 x 5 mm) of the phantom and their concentration (mean Bq/mL) was compared against the concentration in the uniformity ROI and calculated as the following ratio: RC_air/water chamber_ = (chamber concentration (mean Bq/mL))/(uniformity concentration (mean Bq/mL)).

To determine RCs in the cylinders and spill-over effects, the ROI in phantoms were defined with their size matching the contours of the cylinders and not with ROIs larger than the actual size of the cylinders. This decision was chosen for practical reasons in order to closely match the *in vivo* tumor analysis strategy where the regions of interest are drawn on the actual sizes of the tumor. RCs were determined during decay (from 20 to 1 MBq) with and without scatter and attenuation correction for the different isotopes using the 5 to 1 mm cylinders of the phantom. PET/MR RCs were calculated only without correction because the MR does not generate a complete attenuation MAP with the phantoms for proper correction. In addition, ROI analysis according to the standardized NEMA protocol (*2*) was performed as a generic quality control using the automatic tool from the MEDISO quality control software provided with the cameras in which the ROI sizes in the cylinders are double their actual size.

**Table S1.** Cross Calibration (CC) factors between the nanoScan PET/CT or PET/MR scanner and the dose calibrator determined for ^11^C, ^68^Ga, ^18^F, and ^89^Zr with either 3.7 or 20 MBq present in the phantom

| **Isotope** | **Activity (MBq)** | **CC factor PET/CT** | **CC factor PET/MR** |
| --- | --- | --- | --- |
| **^11^C** | 3.7 | 0.95 | 1.02 |
|  | 20 | 0.98 | 1.04 |
| **^68^Ga** | 3.7 | 1.12 | 1.09 |
|  | 20 | 1.13 | 1.09 |
| **^18^F** | 3.7 | 0.96 | 0.92 |
|  | 20 | 0.99 | 0.94 |
| **^89^Zr** | 3.7 | 0.96 | 0.94 |
|  | 20 | 0.98 | 0.94 |

**Table S2.** NanoScan PET/CT and PET/MR recovery coefficients for ^11^C, ^68^Ga,  ^18^F, and ^89^Zr with scatter and attenuation correction (PET/CT) and without (PET/CT and PET/MR) (see Figure 2)

| **PET/CT** | **with correction** |  |  |  |  |  |  |  |  |  |  |  |
| --- | --- | --- | --- | --- | --- | --- | --- | --- | --- | --- | --- | --- |
| **mm** | **^11^C** | | | **^68^Ga** | | | **^18^F** | | | **^89^Zr** | | |
| **5** | 75.9 | ± | 1.4 | 54.1 | ± | 1.5 | 79.7 | ± | 1.2 | 76.6 | ± | 1.4 |
| **4** | 68.8 | ± | 1.7 | 44.7 | ± | 1.0 | 77.2 | ± | 1.7 | 69.3 | ± | 3.2 |
| **3** | 60.3 | ± | 2.1 | 32.9 | ± | 1.4 | 67.6 | ± | 2.7 | 58.7 | ± | 4.2 |
| **2** | 43.1 | ± | 1.3 | 18.6 | ± | 1.0 | 51.8 | ± | 3.1 | 42.3 | ± | 2.9 |
| **1** | 7.9 | ± | 0.9 | 5.1 | ± | 0.7 | 10.6 | ± | 1.4 | 7.8 | ± | 2.6 |
|  |  |  |  |  |  |  |  |  |  |  |  |  |

| **Without correction** | |  |  |  |  |  |  |  |
| --- | --- | --- | --- | --- | --- | --- | --- | --- |
| **mm** | **^11^C** | | **^68^Ga** | | **^18^F** | | **^89^Zr** | |
|  | **PET/CT** | **PET/MR** | **PET/CT** | **PET/MR** | **PET/CT** | **PET/MR** | **PET/CT** | **PET/MR** |
| **5** | 78.4 | 80.6 | 55.9 | 56.0 | 80.3 | 79.8 | 79.7 | 79.5 |
| **4** | 71.5 | 71.8 | 46.6 | 45.4 | 78.5 | 74.0 | 71.1 | 70.9 |
| **3** | 60.7 | 58.3 | 33.8 | 34.4 | 68.5 | 67.3 | 60.9 | 62.7 |
| **2** | 43.3 | 38.2 | 18.9 | 17.9 | 50.9 | 49.6 | 44.5 | 41.7 |
| **1** | 10.1 | 10.6 | 6.5 | 5.9 | 12.5 | 10.6 | 11.4 | 9.1 |

**Table S3.** *Ex vivo* biodistribution of [^18^F]FDG and [^89^Zr]Zr-DFO-NCS-trastuzumab in N87 tumor-bearing nude mice at 70 min and 72 h p.i., respectively. Results are expressed as mean ± SD %IA/g, n=10 mice per group

|  | **[^18^F]FDG (CT mice)** | | | **[^18^F]FDG (MR mice)** | | | **[^89^Zr]Zr-DFO-NCS-trastuzumab**  **(all mice)** | | |
| --- | --- | --- | --- | --- | --- | --- | --- | --- | --- |
|  | **%IA/g** | **±** | **sd** | **%IA/g** | **±** | **sd** | **%IA/g** | **±** | **sd** |
| blood | 0.6 | ± | 0.2 | 0.7 | ± | 0.2 | 7.5 | ± | 1.4 |
| urine | 213.2 | ± | 104.0 | 256.7 | ± | 98.6 | 1.3 | ± | 0.3 |
| skin | 2.9 | ± | 0.6 | 2.9 | ± | 0.7 | 4.4 | ± | 0.7 |
| bladder | 11.5 | ± | 7.3 | 32.9 | ± | 46.2 | 2.9 | ± | 0.4 |
| **tumor** | **3.3** | **±** | **0.7** | **3.1** | **±** | **0.7** | **22.5** | **±** | **3.7** |
| sternum | 3.3 | ± | 1.0 | 2.7 | ± | 0.6 | 2.4 | ± | 0.5 |
| heart | 82.4 | ± | 19.5 | 84.2 | ± | 24.8 | 2.3 | ± | 0.6 |
| lung | 5.6 | ± | 0.7 | 5.6 | ± | 0.9 | 3.6 | ± | 0.5 |
| **liver** | **1.1** | **±** | **0.2** | **1.2** | **±** | **0.2** | **4.9** | **±** | **0.9** |
| pancreas | 1.9 | ± | 0.5 | 1.8 | ± | 0.3 | 0.9 | ± | 0.1 |
| spleen | 5.0 | ± | 0.5 | 5.2 | ± | 1.7 | 3.8 | ± | 1.0 |
| **kidney** | **13.7** | **±** | **3.6** | **13.5** | **±** | **2.0** | **3.6** | **±** | **0.4** |
| muscle | 1.0 | ± | 0.6 | 0.8 | ± | 0.3 | 0.7 | ± | 0.1 |
| thigh-bone | 2.0 | ± | 0.4 | 1.7 | ± | 0.4 | 2.1 | ± | 0.4 |
| colon | 4.7 | ± | 1.5 | 4.9 | ± | 0.8 | 1.6 | ± | 0.9 |
| ileum | 4.4 | ± | 1.1 | 3.9 | ± | 0.7 | 1.7 | ± | 0.6 |
| stomach | 1.5 | ± | 0.6 | 1.5 | ± | 0.3 | 0.7 | ± | 0.3 |
| **brain** | **5.0** | **±** | **1.1** | **4.8** | **±** | **0.4** | **0.3** | **±** | **0.1** |
| head (rest) | 3.9 | ± | 0.6 | 4.0 | ± | 0.6 | 2.2 | ± | 0.3 |
| knee | 1.9 | ± | 0.8 | 2.3 | ± | 0.7 | 4.8 | ± | 1.0 |
| tail | 6.7 | ± | 4.7 | 6.8 | ± | 5.4 | 2.0 | ± | 0.5 |

**Table S4.** N87 tumor sizes assessed by PET imaging ROIs or by weight in *ex vivo* biodistribution studies

|  | **Range** | **Average ± sd** | **Correlation (R^2^)** |
| --- | --- | --- | --- |
| **^18^F study- PET/CT mice (n=10)** ROI (mm^3^) | 75-532 | 238 ± 117 | **0.90** |
| Weight (mg) | 75-590 | 225 ± 127 |  |
| **^18^F study- PET/MR mice (n=10)** ROI (mm^3^) | 53-404 | 169 ± 87 | **0.88** |
| Weight (mg) | 78-428 | 224 ± 104 |  |
| **^89^Zr study (n=10)** ROI (mm^3^)-CT | 43-152 | 110 ± 30 | **0.84** |
| ROI (mm^3^)-MR | 43-147 | 105 ± 31 | **0.75** |
| Weight (mg) | 57-207 | 126 ± 39 |  |

**Table S5.** Average and individual PET/CT and PET/MR imaging results of [^18^F]FDG and [^89^Zr]Zr-DFO-NCS-trastuzumab in N87 tumor-bearing nude mice at 70 min and 72 h p.i., respectively. Results are expressed as mean ± SD %IA/g, n=10 mice per group. Individual PET/CT and PET/MR imaging results are presented with their corresponding *ex vivo* biodistribution result per animal (in %IA/g) for the different organs evaluated (tumor, brain, kidney and liver). Those results were further used for ratios and Bland-Altman plots. No data were excluded except for obvious reasons*.

|  | **[^18^F]FDG (CT mice)** | | | **[^18^F]FDG (MR mice)** | | | **[^89^Zr]Zr-DFO-NCS-trastuzumab**  **(CT)** | | | **[^89^Zr]Zr-DFO-NCS-trastuzumab**  **(MR)** | | |
| --- | --- | --- | --- | --- | --- | --- | --- | --- | --- | --- | --- | --- |
|  | **%IA/g** | **±** | **sd** | **%IA/g** | **±** | **sd** | **%IA/g** | **±** | **sd** | **%IA/g** | **±** | **sd** |
| **tumor** | 2.9 | ± | 0.4 | 2.8 | **±** | 0.5 | 16.7 | ± | 2.9 | 17.5 | **±** | 2.7 |
| **liver** | 1.4 | ± | 0.3 | 1.5 | **±** | 0.4 | 5.0 | ± | 0.8 | 5.7 | **±** | 1.1 |
| **kidney** | 11.4 | ± | 2.3 | 12.0 | **±** | 1.6 | 3.1 | ± | 0.4 | 3.3 | **±** | 0.4 |
| **brain** | 5.4 | ± | 1.1 | 4.9 | **±** | 0.6 | 0.8 | ± | 0.1 | 1.0 | **±** | 0.2 |

| **Tumors** | **(%IA/g)** |  |  |  |  |  |  |
| --- | --- | --- | --- | --- | --- | --- | --- |
| **^18^F-CT** | | **^18^F-MR** | | **^89^Zr-CT** | | **^89^Zr-MR** | |
| **PET** | **ex vivo** | **PET** | **ex vivo** | **PET** | **ex vivo** | **PET** | **ex vivo** |
| 2.49 | 3.05 | 3.58 | 4.59 | 21.38 | 25.73 | 23.02 | 25.73 |
| 2.50 | 3.08 | 3.07 | 3.43 | 17.09 | 23.07 | 17.46 | 23.07 |
| 2.93 | 2.84 | 2.06 | 2.92 | 21.36 | 28.54 | 22.89 | 28.54 |
| 2.80 | 3.17 | 2.11 | 2.61 | 17.18 | 24.09 | 19.72 | 31.12 |
| 3.04 | 4.17 | 2.12 | 2.34 | 19.77 | 25.06 | 18.01 | 24.09 |
| 2.72 | 3.60 | 2.46 | 2.46 | 18.05 | 23.14 | 19.09 | 25.06 |
| 3.45 | 3.80 | 2.03 | 2.10 | 19.16 | 23.42 | 17.36 | 21.84 |
| 3.50 | 3.60 | 2.18 | 2.58 | 17.15 | 22.67 | 17.89 | 23.63 |
| 3.27 | 4.35 | 2.82 | 3.85 | 16.37 | 22.43 | 15.57 | 23.14 |
| 3.14 | 3.86 | 3.23 | 3.56 | 17.44 | 22.47 | 18.53 | 23.42 |
| 2.78 | 2.56 | 2.88 | 2.96 | 16.69 | 22.83 | 18.10 | 22.67 |
| 3.28 | 3.69 | 3.02 | 2.91 | 16.84 | 21.30 | 18.46 | 22.43 |
| 2.43 | 2.80 | 2.49 | 2.37 | 14.02 | 19.20 | 18.64 | 22.47 |
| 2.74 | 3.60 | 2.58 | 2.81 | 12.40 | 17.40 | 17.57 | 22.83 |
| 2.24 | 2.33 | 2.78 | 2.77 | 12.96 | 16.66 | 17.68 | 21.30 |
| 2.18 | 2.28 | 3.02 | 3.16 | 12.70 | 17.33 | 14.13 | 19.20 |
| 2.38 | 2.50 | 3.67 | 4.87 | 12.83 | 17.03 | 13.72 | 17.40 |
| 2.75 | 2.53 | 3.46 | 3.24 |  |  | 13.73 | 16.66 |
| 3.49 | 4.06 |  |  |  |  | 14.42 | 17.33 |
| 2.95 | 4.11 |  |  |  |  | 13.13 | 17.03 |
|  |  |  |  |  |  |  |  |
| **Brain** | **(%IA/g)** |  |  |  |  |  |  |
| **^18^F-CT** | | **^18^F-MR** | | **^89^Zr-CT** | | **^89^Zr-MR** | |
| **PET** | **ex vivo** | **PET** | **ex vivo** | **PET** | **ex vivo** | **PET** | **ex vivo** |
| 4.30 | 3.91 | 5.55 | 5.12 | 0.95 | 0.39 | 1.09 | 0.39 |
| 4.64 | 4.36 | 5.18 | 5.45 | 1.01 | 0.39 | 1.42 | 0.39 |
| 4.92 | 4.59 | 3.83 | 4.20 | 0.70 | 0.21 | 1.15 | 0.21 |
| 6.04 | 5.54 | 5.49 | 4.92 | 0.84 | 0.28 | 1.06 | 0.28 |
| 7.95 | 7.58 | 4.34 | 4.74 | 0.79 | 0.32 | 1.12 | 0.28 |
| 6.12 | 5.70 | 5.23 | 4.82 | 0.91 | 0.25 | 1.14 | 0.32 |
| 5.13 | 4.61 | 5.23 | 4.81 | 0.77 | 0.24 | 0.77 | 0.25 |
| 4.60 | 4.23 | 5.20 | 4.36 | 0.55 | 0.24 | 0.80 | 0.24 |
| 5.16 | 5.11 | 4.45 | 4.72 | 0.75 | 0.25 | 0.61 | 0.24 |
| 4.72 | 4.47 |  |  |  |  | 0.79 | 0.25 |
|  |  |  |  |  |  |  |  |
| **Kidney** | **(%IA/g)** |  |  |  |  |  |  |
| **^18^F-CT** | | **^18^F-MR** | | **^89^Zr-CT** | | **^89^Zr-MR** | |
| **PET** | **ex vivo** | **PET** | **ex vivo** | **PET** | **ex vivo** | **PET** | **ex vivo** |
| 11.54 | 14.80 | 10.02 | 10.07 | 3.92 | 3.68 | 3.73 | 3.68 |
| 10.12 | 11.53 | 11.09 | 14.78 | 3.14 | 3.54 | 3.35 | 3.54 |
| 9.96 | 10.43 | 11.19 | 16.04 | 2.78 | 3.47 | 2.85 | 3.47 |
| 15.98 | 17.90 | 11.41 | 12.27 | 2.95 | 4.25 | 3.67 | 3.67 |
| 9.35 | 12.97 | 14.62 | 16.72 | 3.50 | 4.21 | 3.17 | 4.25 |
| 13.05 | 13.93 | 11.50 | 12.54 | 2.70 | 3.29 | 3.71 | 4.21 |
| 14.31 | 21.29 | 14.28 | 14.01 | 3.18 | 3.39 | 2.65 | 3.29 |
| 9.75 | 10.22 | 12.90 | 13.42 | 2.80 | 3.20 | 3.39 | 3.39 |
| 9.94 | 10.48 | 11.17 | 12.66 | 2.58 | 3.09 | 2.84 | 3.20 |
| 10.46 | 13.84 |  |  |  |  | 3.27 | 3.09 |
|  |  |  |  |  |  |  |  |
| **Liver** | **(%IA/g)** |  |  |  |  |  |  |
| **^18^F-CT** | | **^18^F-MR** | | **^89^Zr-CT** | | **^89^Zr-MR** | |
| **PET** | **ex vivo** | **PET** | **ex vivo** | **PET** | **ex vivo** | **PET** | **ex vivo** |
| 1.15 | 0.90 | 2.28 | 1.72 | 6.11 | 5.38 | 6.06 | 5.38 |
| 1.82 | 1.37 | 0.96 | 1.03 | 5.99 | 5.56 | 6.32 | 5.56 |
| 1.63 | 1.35 | 1.13 | 1.12 | 5.18 | 5.23 | 5.47 | 5.23 |
| 1.69 | 1.28 | 1.38 | 1.16 | 4.03 | 3.95 | 8.10 | 6.88 |
| 1.84 | 1.45 | 1.48 | 1.16 | 4.13 | 3.90 | 4.50 | 3.95 |
| 1.17 | 0.98 | 1.58 | 1.22 | 4.52 | 4.28 | 4.93 | 3.90 |
| 1.25 | 1.03 | 1.55 | 1.16 | 5.08 | 4.69 | 4.50 | 4.28 |
| 1.17 | 1.13 | 1.58 | 1.26 | 4.70 | 4.57 | 5.52 | 4.69 |
| 1.08 | 0.94 | 1.56 | 1.14 | 5.63 | 5.03 | 5.19 | 4.57 |
| 1.16 | 0.99 |  |  |  |  | 6.07 | 5.03 |

*Data excluded: brain (n=1 value), out of field of view during PET/MR data acquisition in the ^18^F study, tumor (n=1 value) shape not possible to delineate accurately on PET/CT in the ^89^Zr study, one animal in the ^18^F study (n = 1, PET/MR group) and in the ^89^Zr study (n= 1, PET/CT acquisition) because the data were not acquired properly during imaging.


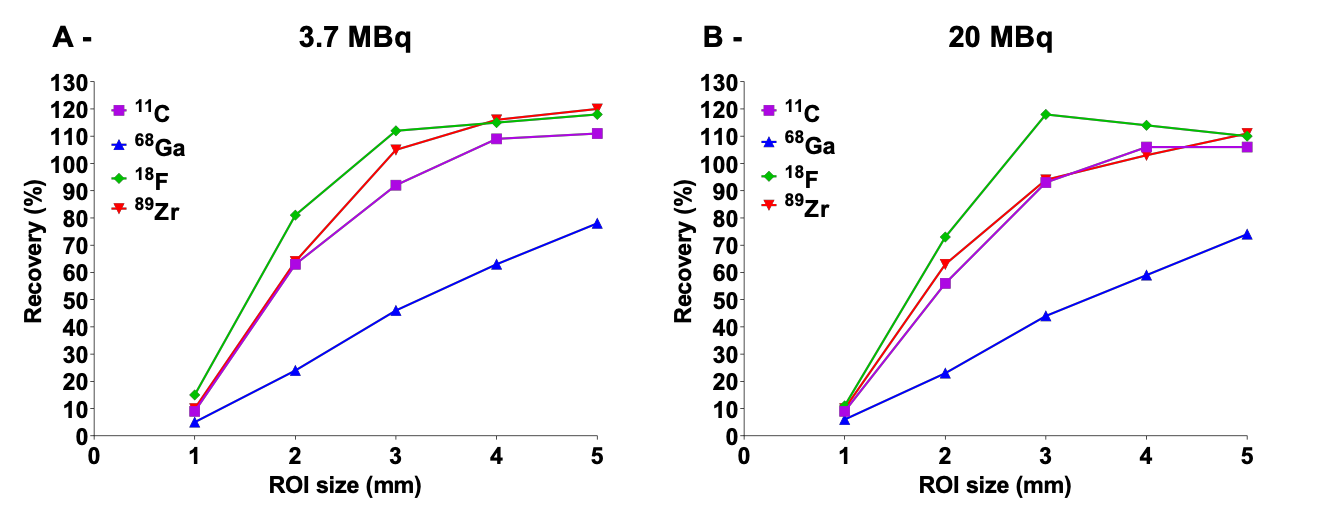


**Figure S1**. NanoScan PET/CT recovery coefficients for ^11^C, ^68^Ga, ^18^F, and ^89^Zr at 3.7 **(A)**, and 20 MBq **(B)**. Data obtained using the Mediso software using the TeraTomo reconstruction algorithm and according to the NEMA standardized recommendations

**
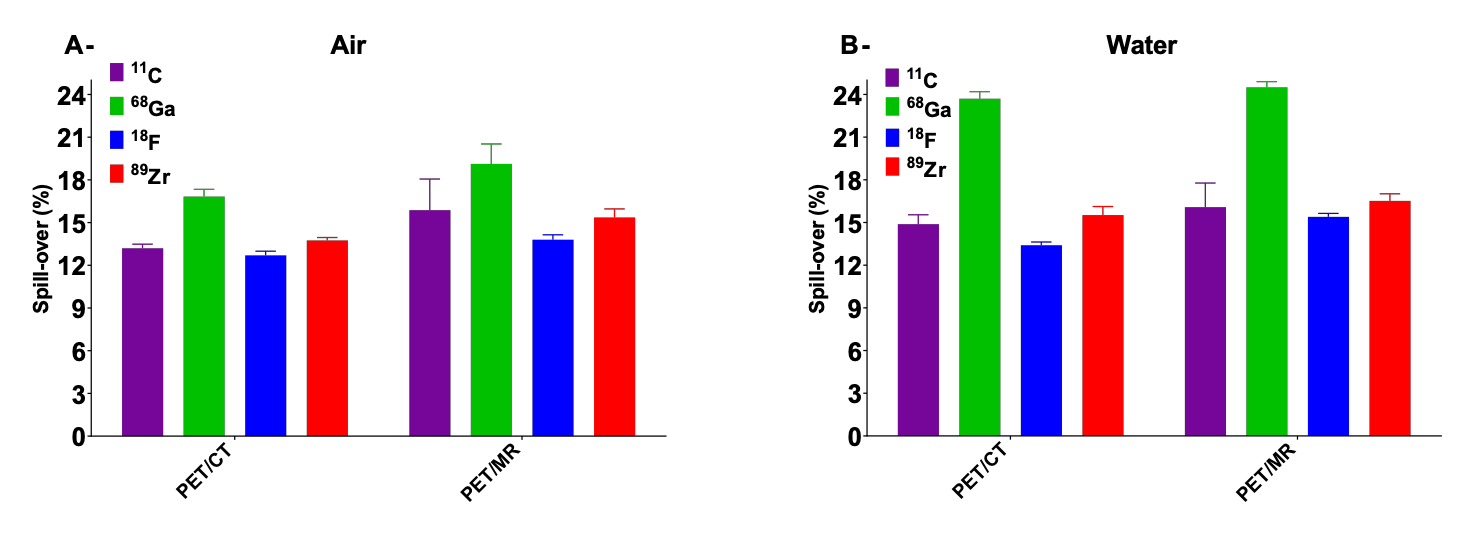
**

**Figure S2.** Comparison of spill-over effects in air **(A)** and in water **(B)** between PET/CT and PET/MRI, for ^11^C, ^68^Ga, ^18^F, and ^89^Zr


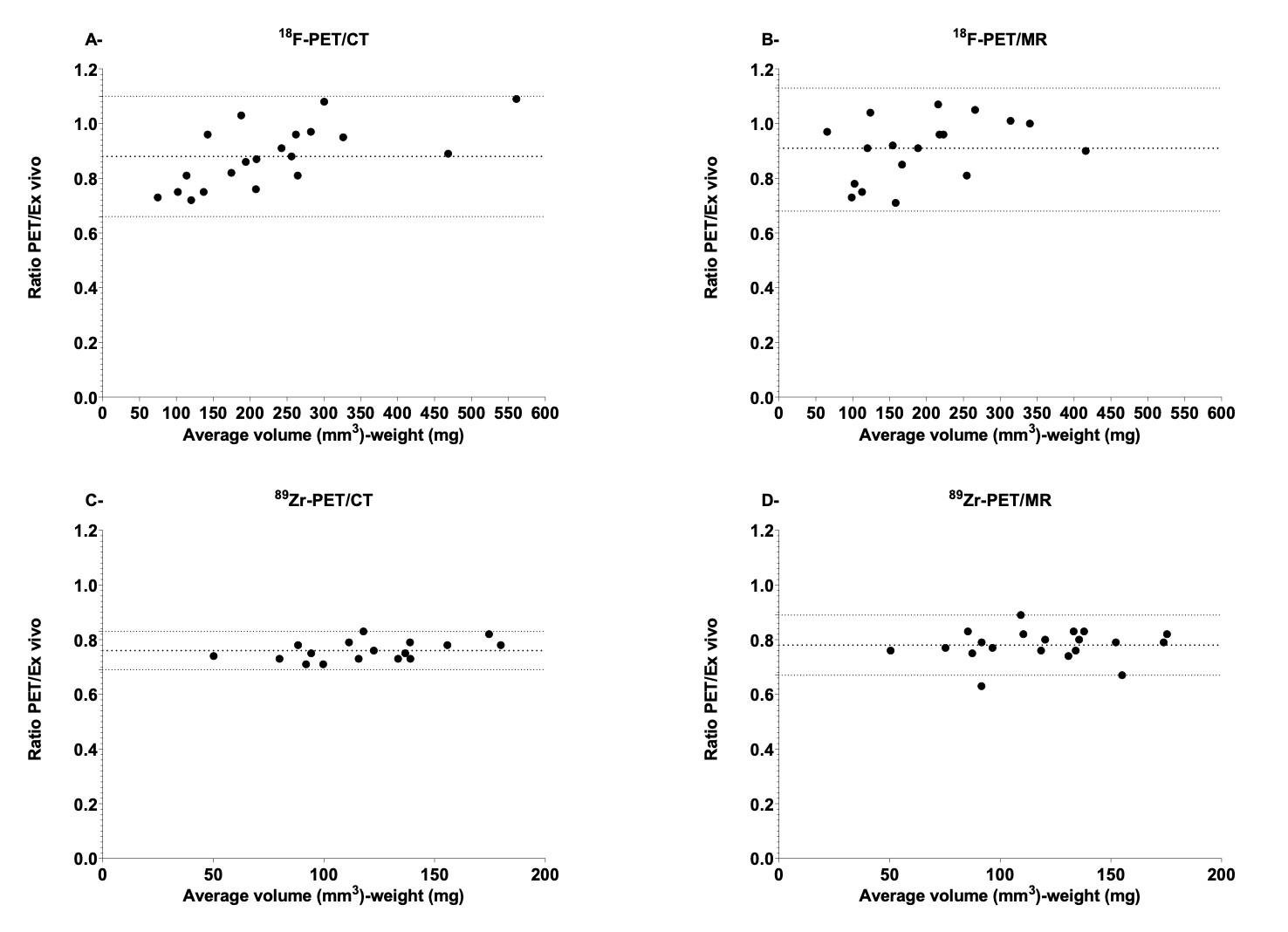


**Figure S3.** Plots comparing tumor uptake of [^18^F]FDG (**A,B**) and [^89^Zr]Zr-DFO-NCS-trastuzumab (**C,D**) assessed by PET imaging (PET/CT: **A,C**; PET/MRI: **B,D**) or by *ex vivo* biodistribution in relation to average tumor volume-weight. The middle dotted line shows the Bias (mean of the ratio PET/*ex vivo* biodistribution) and the upper and lower dotted lines show the 95% limits of agreement. Average corresponds to the average tumor size value per animal between PET (mm^3^) and *ex vivo* biodistribution (mg)

**
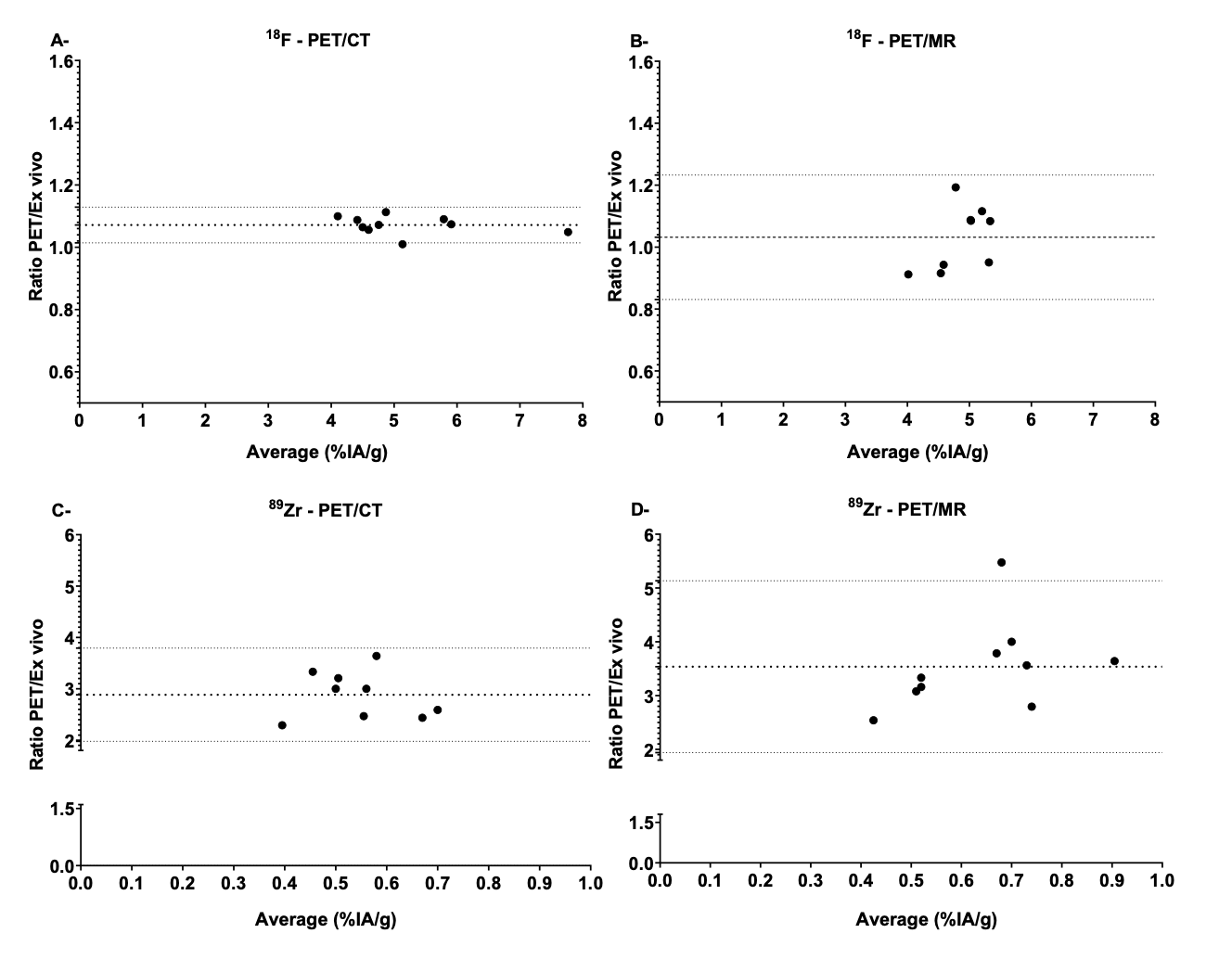
**

**Figure S4.** Bland-Altman plots comparing brain uptake of [^18^F]FDG (**A,B**) and [^89^Zr]Zr-DFO-NCS-trastuzumab (**C,D**) assessed by PET imaging (PET/CT: **A,C**; PET/MRI: **B,D**) or by *ex vivo* biodistribution. The middle dotted line shows the Bias (mean of the ratios) and the upper and lower dotted lines show the 95% limits of agreement. Average (%IA/g) corresponds to the average uptake value per animal between PET and *ex vivo* biodistribution

**
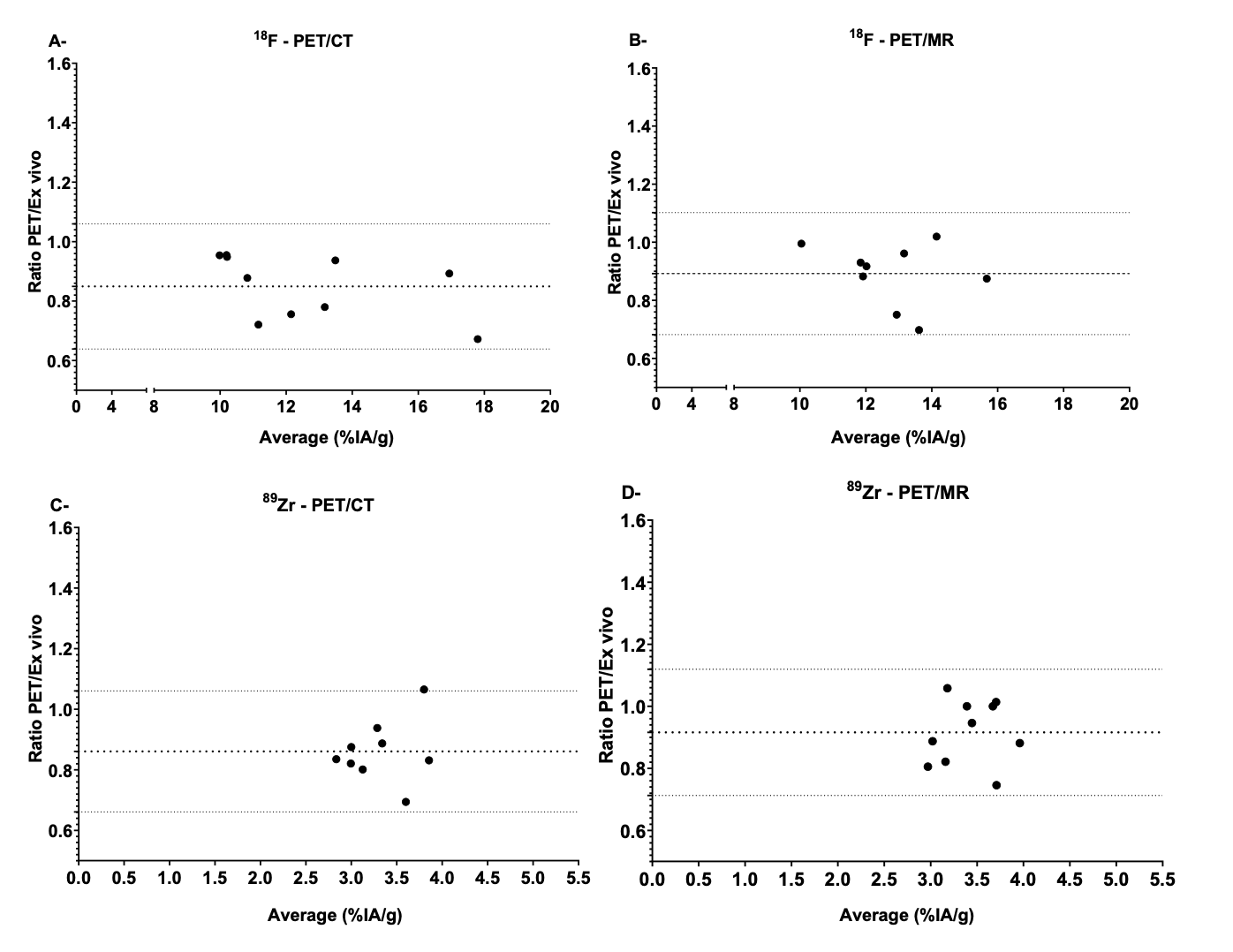
**

**Figure S5.** Bland-Altman plots comparing kidney uptake of [^18^F]FDG (**A,B**) and [^89^Zr]Zr-DFO-NCS-trastuzumab (**C,D**) assessed by PET imaging (PET/CT: **A,C**; PET/MRI: **B,D**) or by *ex vivo* biodistribution. The middle-dotted line shows the Bias (mean of the ratios) and the upper and lower dotted lines show the 95% limits of agreement. Average (%IA/g) corresponds to the average uptake value per animal between PET and *ex vivo* biodistribution


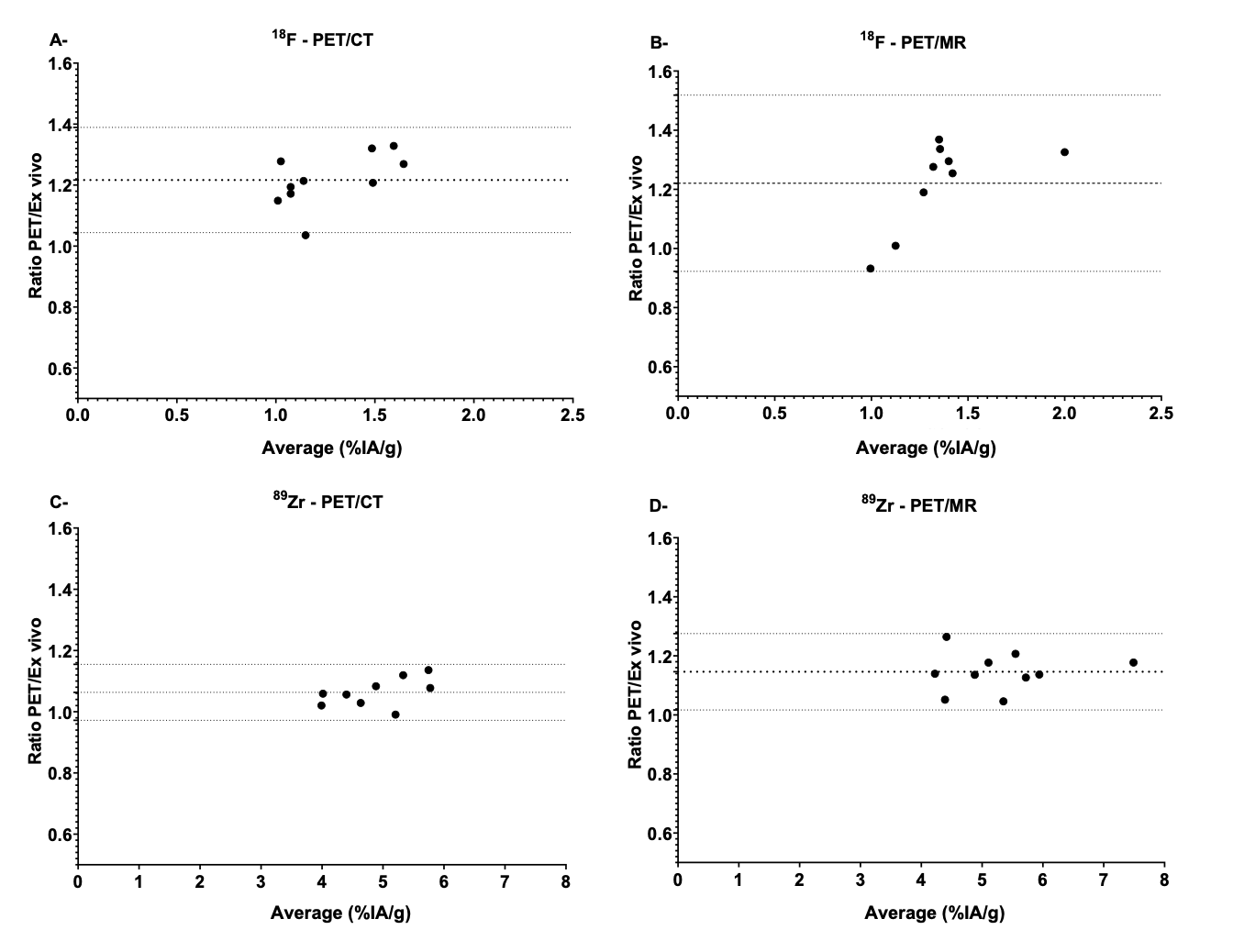


**Figure S6.** Bland-Altman plots comparing liver uptake of [^18^F]FDG (**A,B**) and [^89^Zr]Zr-DFO-NCS-trastuzumab (**C,D**) assessed by PET imaging (PET/CT: **A,C**; PET/MRI: **B,D**) or by *ex vivo* biodistribution. The middle-dotted line shows the Bias (mean of the ratios) and the upper and lower dotted lines show the 95% limits of agreement. Average (%IA/g) corresponds to the average uptake value per animal between PET and *ex vivo* biodistribution

**References**

1. Kaalep A, Huisman M, Sera T, Vugts D, Boellaard R. Feasibility of PET/CT system performance harmonisation for quantitative multicentre 89Zr studies. *EJNMMI Phys*. 2018;5:26.

2. National Electrical Manufacturers Association. NEMA Standards Publication NU 4-2008 Performance Measurements of Small Animal Positron Emission Tomographs. Rosslyn, VA: National Electrical Manufacturers Association.; 2008.
